# An analysis of organism lifelines in an industrial bioreactor using Lattice‐Boltzmann CFD

**DOI:** 10.1002/elsc.202100159

**Published:** 2022-03-16

**Authors:** Cees Haringa

**Affiliations:** ^1^ Bioprocess Engineering Biotechnology Department Delft University of Technology Delft the Netherlands

**Keywords:** CFD, Euler‐Lagrange, fermentation, Lattice Boltzmann, lifeline analysis

## Abstract

Euler‐Lagrange CFD simulations, where the biotic phase is represented by computational particles (parcels), provide information on environmental gradients inside bioreactors from the microbial perspective. Such information is highly relevant for reactor scale‐down and process optimization. One of the major challenges is the computational intensity of CFD simulations, especially when resolution of dynamics in the flowfield is required. Lattice‐Boltzmann large‐eddy simulations (LB‐LES) form a very promising approach for simulating accurate, dynamic flowfields in stirred reactors, at strongly reduced computation times compared to finite volume approaches. In this work, the performance of LB‐LES in resolving substrate gradients in large‐scale bioreactors is explored, combined with the inclusion of a Lagrangian biotic phase to provide the microbial perspective. In addition, the hydrodynamic performance of the simulations is confirmed by verification of hydrodynamic characteristics (radial velocity, turbulent kinetic energy, energy dissipation) in the impeller discharge stream of a 29 cm diameter stirred tank. The results are compared with prior finite volume simulation results, both in terms of hydrodynamic and biokinetic observations, and time requirements.

**Abbreviations**
CFD: computational fluid dynamics
DNS: direct numerical simulation
EL: Euler‐Lagrange
FV: finite volume
GPU: graphical processing unit
LB: Lattice Boltzmann
LES: large‐eddy simulation
RANS: Reynolds‐Averaged Navier‐Stokes

## 1 INTRODUCTION

Computational Fluid Dynamics (CFD) simulations are increasingly popular for the analysis of bioprocess performance, for up‐ and downscaling of bioprocesses, and process optimization. In recent years, the usage of so‐called Euler‐Lagrange CFD simulations, where clusters of micro‐organism are tracked as computational particles (parcels) in order to analyze bioprocesses from the microbial viewpoint, has gained popularity. This approach is used to collect so‐called lifelines [1, 2] representing time‐series of the environmental conditions encountered by cells in bioreactors featuring heterogeneous environments. Lifeline analysis [3] is particularly useful for scale‐down simulators design [4], aiming to mimic the environmental dynamics encountered by cells in lab‐scale equipment. Euler‐Lagrange simulations coupled with metabolic models [1, 2, 5, 6, 7] are furthermore interesting for predicting changes in the metabolic response to environmental dynamics, and the effect thereof on overall process performance, for in‐silico process scale‐up and optimization [7].

However, the required runtime for CFD hampers routine application. Thus far, the finite volume method (FV), particularly using Reynolds‐Averaged Navier Stokes (RANS) turbulence models, is the workhorse of CFD. While the ability to produce steady‐state, averaged flowfields can keep computation times tractable, this often leads to the omission of dynamics relevant for bioreactor performance, such as the impact of macro‐oscillations on mixing [8, 9, 10] and gas‐plume oscillations [11, 12]. FV‐RANS simulations that do capture these dynamics have strongly prohibitive computation times, in the order of 1 week computation time per minute flow time, and simulations including large numbers of parcels may require up to a month of computation, even assuming steady‐state flow [3]. Such computational expenses lead to CFD being mostly used for troubleshooting or assessment of a few design alternatives, rather than routine usage in process design, let alone tracking microbial lifelines for extended periods.

There hence is a strong incentive to develop spatially‐resolved bioprocess simulations with vastly reduced computation time. For some applications, lower resolution compartment models [13, 14, 15, 16, 17] or strategies that reconstruct flowfields, such as rCFD [18] or machine learning [19, 20, 21], may be a feasible alternative. However, for example, design optimization these may not (yet) be feasible, and may anyhow require CFD simulations for training/calibration, meaning there is a need for faster CFD simulations, in particular when resolved flow dynamics are relevant.

**PRACTICAL APPLICATION**

CFD simulations are gaining popularity as a tool for bioprocess analysis, especially in the context of scale‐down/scale‐up and process performance analysis. Usage of particle tracking methods to compute so‐called organism lifelines, in order to analyze the bioprocess from the microbial viewpoint, are especially interesting. This research shows the suitability of the Lattice‐Boltzmann CFD simulation method for the analysis of bioreactors from the microbial viewpoint. Compared to finite‐volume CFD simulations, the computation time requirements of the method are vastly reduced, while the fully dynamic Lattice Boltzmann method increases the accuracy in terms of turbulence and mixing characteristics. The improved accuracy and reduced runtime make routine application of lifeline analysis for industrial bioreactors, towards design of scale‐down simulators or towards estimating the impact of heterogeneous conditions on yield/production rate, feasible.

An approach that has challenged FV methods regarding speed and accuracy for many years is Lattice Boltzmann (LB) [22, 23, 24, 25, 26]. While promising mixing results were obtained already 15 years ago due to the inherently dynamic large‐eddy simulation (LES) formulation [27], adoption in applied research and industry has, to date, been limited. Possibly, this is because until recently commercial/open‐source LB codes were lacking, and in‐house code development forms a substantial hurdle for most users. Furthermore, some relevant physical phenomena were lacking until recently, and the need for computing clusters may have been prohibitive. In recent years, this has been changing; a more extensive range of physical phenomena has been included in LB, including reaction, particle/bubble flow [28, 29, 30], rheology [31], and mass transfer [32]. In addition, GPU‐based LB [33] has brought hardware requirements within reach for a wider range of users, while open‐source and commercial codes have simplified application [24, 32].

The objective of this paper is to show that LB‐LES methods can provide highly resolved *dynamic* CFD simulations of bioreactors, including biokinetics and microbial lifeline tracking, with computation times similar to those of steady‐state FV‐RANS, and much below dynamic FV‐RANS and LES. This unlocks more routine usage of CFD for bioreactor analysis and optimization, without requiring high‐performance computing clusters. While the hydrodynamic fidelity of LB simulations is well established, the hydrodynamics of the impeller discharge stream are studied to verify the current setup, and to benchmark performance versus both FV simulations. Mixing is verified with experimental data, and the manifestation of substrate gradients is studied. Next, for the first time, microbial lifeline analysis is explored in the LB‐framework; lifeline statistics and the intracellular metabolite response are compared with prior FV‐RANS results. To conclude, time requirements of LB‐LES simulations are discussed.

## 2 MATERIALS AND METHODS

The LB‐LES simulations are conducted using M‐Star CFD 3.3.36 (M‐Star Simulations, LLC). All simulations are conducted on a XEON‐W2265 desktop using a NVIDIA RTX3090 24GB GPU for GPU‐based computing. Two case‐studies are considered. First, the single‐phase hydrodynamics are benchmarked for fully turbulent flow with a single Rushton turbine [34, 35], a case for which experimental velocity [9, 36], kinetic energy [9, 37, 38], and energy dissipation data [39, 40] are available; they are furthermore compared with FV‐RANS and FV‐LES simulations, reported previously [9, 10]. Some of the FV simulations are partially re‐run on the XEON‐W2265 desktop using ANSYS FLUENT 2022 R1 to provide a contemporary benchmark for the computational expense. Second, the penicillin 54 m^3^ bioreactor case is considered, in terms of mixing, substrate gradient, and microbial lifelines, and compared to prior FV‐RANS results [3].

### 2.1 Simulation setup

*M‐Star* Lattice‐Boltzmann uses the with the D3Q19 velocity vector set for discretization of the velocities; for details regarding the LB method, we refer to the M‐Star documentation [41] or prior technical papers on LB [23, 42]. The equations are solved on a constant‐spaced lattice, defined by the number of divisions NX in the horizontal x‐direction (extend equal to tank diameter *T*); various NX are used to quantify the influence of spatial resolution. Walls and moving bodies are implemented using the immersed boundary method [43]. The boundary is grid‐aligned; although an interpolated boundary is more accurate by avoiding stair‐stepping, no impact was observed in test simulations. All walls are no‐slip surfaces; the top a no‐shear surface to model a filled tank without resolving the free surface. All simulations are conducted with the Smagorinsky subgrid model using $C_{LES}=0.1$ . [25]

### 2.2 1‐impeller case

The 1‐impeller uses the geometry of Jahoda et al. [34]. The flat‐bottom tank has diameter T=0.29 m and (liquid) height $H_{l}=T$, containing a 6‐blade Rushton impeller with diameter D=T/3 and off‐bottom clearance C=T/3. The tank contains four baffles over the full height, with width T/10. For FV‐RANS simulations, inclusion of the impellers as 3D bodies resulted in under‐estimation of the energy dissipation rate ε [44, 45]; 2D sheet bodies gave better predictions [46, 47, 48]. Both 2D sheet bodies and 3D internal bodies are used in this study, for comparison. In case of 3D bodies, the baffle and impeller blade thickness are 5 mm. The agitation rate is *N* = 300 RPM. The density and viscosity are set to ρ=1000 kg/m^3^ and $\mu_{l}=0.001$ Pa s, respectively. Hydrodynamic performance is evaluated by quantifying radial velocity $U_{rad},$ turbulent kinetic energy $k_{t}$ and energy dissipation rate ε in the impeller discharge stream., at height *y = C*, over a line rotated 45^o^ with respect to the baffle plane. Additionally, the power number based on torque ($Po_{\tau }$) and on ∫εdV, the integral energy dissipation ($Po_{\varepsilon })$ is evaluated. M‐STAR features the option of wall functions to reduce the computational burden in case high‐accuracy resolution of near‐wall flow is not required, as is the case. Wall functions reduce the over‐estimation of energy dissipation in the wall region [49]. The Werner and Wengle approach [50] is implemented in M‐STAR. Although not the most accurate, it uses a direct rather than iterative approach to calculate wall shear‐stress, which was found favorable. The impact of using wall functions is tested in this work. Furthermore, the timestep size is varied by changing the Courant number $Co=v_{tip}\;\cdot \Delta t/\Delta x$. An overview of all simulations is provided in Table 1.

**TABLE 1:** Overview of the single impeller cases simulated in this study

Case	X‐divisions	Wall function	Impeller	Courant no.
180‐BASE	180	NO	3D	0.05
180‐WF	180	YES	3D	0.05
180‐LC	180	NO	3D	0.01
180‐LCWF	180	YES	3D	0.01
180‐2D	180	NO	2D	0.05
180‐2DWF	180	YES	2D	0.05
360‐BASE	360	NO	3D	0.05
360‐WF	360	YES	3D	0.05
360‐LC	360	NO	3D	0.01
360‐2D	360	NO	2D	0.05

Categories: WF = wall function, Impeller = geometry type, Co = Courant number.

All simulations run for 60 s flow‐time; 20 s are used to establish a pseudo‐steady flow profile, subsequently the flow‐ and turbulence fields as well as power numbers are time‐averaged for 40 s (ca. 6 mixing times). Smooth averaged discharge profiles are observed, although longer flow‐times would be needed to fully account for all macro‐instabilities [33]. To conclude, the density is a computed parameter in LB, and large fluctuations in density reduce the accuracy of the method. Therefore, the so called lattice‐density $\rho_{LB}$ has been monitored; lattice‐density fluctuations of ca. 1% are acceptable, typically requiring Co≈0.05 in stirred applications [24].

### 2.3 Bioreactor case

A 54 m^3^ penicillin production reactor is considered [3, 7]. The flat‐bottom tank has diameter T=3 m and (liquid) height $H_{l}=7.7$ m. The tank contains an 8‐blade Rushton at off‐bottom clearance C=0.9 m and 6‐blade Rushton with mutual clearance ΔC=3.0 m, both with D=1.3 m. The shaft diameter is $d_{s}=0.27$ m. Four baffles are installed over the full height, with baffle width T/10. The agitation rate is set to N=98 RPM. The density and viscosity are set to ρ=1000 kg/m^3^ and $\mu_{l}=0.001$ Pa s. Based on the single‐impeller case, the simulations are conducted with 2D sheet‐body internals without wall functions. Due to the limited fluctuations in $\rho_{LB}$ observed in the 1‐impeller case, the Courant number is set to Co=0.075, to speed up the simulations without significantly reducing accuracy.

#### 2.3.1 Mixing simulations

Mixing simulations are conducted using the scalar transport model in M‐STAR, which models a scalar passively transported with the flow, using the general scalar transport equation, Equation (1).

(1) $$\frac{\partial C_{s}}{\partial t}=\nabla \cdot \left(D\nabla C_{s}\right)-\nabla \cdot \left(uC_{s}\right)$$

The equation is solved using a flux‐conserving van Leer scheme; no treatment for subgrid turbulent diffusion is implemented currently. Mixing simulations are conducted at three different resolutions: NX=120,180,300; the cases named L‐120, L‐180, and L‐300, respectively. These simulations run for 60 s (ca. 1 mixing time) before tracer injection to establish pseudo‐steady flow. At t=60 s an instantaneous tracer injection of 0.5 mole/L is performed in a spherical volume of 0.4 m diameter, at y=7.35 m, r=0.8 m off‐center, in the baffle plane $\left(\theta =0^{o}\right)$, matching prior FV‐RANS simulations [3]. The tracer has diffusion coefficient $D=6\cdot 10^{-10}$ m^2^/s. To facilitate comparison with prior work, mixing is monitored with a single‐point probe at the bottom (y=0.25 m, r=0.75 m off‐center, $\theta =180^{o}$). In contrast to the frozen‐flow FV‐RANS simulations, LB‐LES is inherently dynamic. M‐STAR does feature a frozen‐flow option using the time‐averaged flowfield; as this may benefit analysis of gradient formation/parcel tracking over long timespans, mixing performance with this option is assessed, using the coarsest mesh (NX‐120). As LB density fluctuations are no issue once the flow is frozen, the timestep size can be increased. Test are conducted using Co=0.075, Co=0.375, and Co=0.75, designated as FF‐1, FF‐5, FF‐10, in the frozen‐flow phase. The flow is first established for 60 s, then time‐averaged for 180 s. At t=240 s the flow is frozen, and at t=250 s tracer is injected using the same procedure as before. Table 2 lists an overview of all large‐bioreactor cases.

**TABLE 2:** Overview of large‐scale bioreactor cases simulated in this study

Case	X‐div.	Flowtime	No. Parcels ($N_{p})$	Courant	Frozen flow
L‐120	120	240	none	0.075	NO
L‐180	180	240	none	0.075	NO
L‐300	300	240	none	0.075	NO
L‐FF1	120	240	none	0.075 (frozen flow)	YES
L‐FF5	120	240	none	0.375 (frozen flow)	YES
L‐FF10	120	240	none	0.75 (frozen flow)	YES
L‐120‐PT	120	2000	2500	0.075	NO

#### 2.3.2 Substrate uptake

Substrate uptake is modeled using Monod kinetics ( $q_{s}=q_{s,max}\;C_{s}/(K_{s}+C_{s}$)), coupled to the extracellular glucose concentration field. The reaction terms is calculated using the local substrate concentration $C_{s}$; the change in scalar concentration due to reaction is computed in a step separate from scalar transport (Equation 1), using explicit 4^th^ order Runge‐Kutta integration; since the typical timestep size is of O(μs) and the reaction timescale of O(ms), there are no issues with the use of an explicit scheme [41]. A biomass concentration of $C_{x}=55$ g_dw_ /kg is assumed, with a maximum specific consumption rate $q_{s,max}=1600\cdot 10^{-6}$ mol/g_dw_/h and affinity $K_{s}=7.8\cdot 10^{-6}$ mol/kg [51]. The glucose feed rate is set to $F_{s}=0.37$ mol/s, using the injection volume used in the mixing study. An initial concentration of $C_{s,init}=7.8\cdot 10^{-5}$ mol/kg $\left(10\;K_{s}\right)$ is set throughout the reactor. To compare the manifested gradients with prior steady‐state FV‐RANS, time‐averaging is applied for 1900 s, simultaneous with parcel tracking, discarding the first 100 s to remove transient effects.

#### 2.3.3 Parcel tracking

A total of 2500 parcels is tracked over a span of 2000 s, using the coarsest lattice (NX = 120). The parcels are injected in a volume (diameter 0.2 m) at y=3 *m*, r=0.5 m off‐center, $\theta =0^{o}$, at *t* = 0. A local injection rather than full domain injection is chosen to avoid release of parcels within the immersed boundary representing the impellers. While this does require ca. 60 s to disperse the particles, the additional burden is very limited. The parcels are considered massless, representing micron‐sized cells with Stokes numbers close to 0; hence, they immediately adapt to the local liquid velocity. For each parcel, the scaled local substrate uptake rate $q_{s}/q_{s,max}$ is registered every $\Delta t_{p}=0.03$s. The registered data for each parcel at every registered timestep is stored in paraview files, which are exported as .csv files and reordered into lifelines ($q_{s}/q_{s,max}$ vs. t per particle) using Julia 1.6 (https://julialang.org/). The first 100 s of each lifeline are discarded to account for the above‐mentioned dispersion. The lifelines stored in .csv format and transferred to MATLAB 2021b for further analysis.

#### 2.3.4 Lifeline analysis

The lifelines are subject to regime‐ and arc‐analysis [3], briefly summarized below. In regime analysis, the broth is divided into a number of metabolic regimes, based on the local biomass specific uptake rate $q_{s}$. The starvation (S) regime is defined as $\frac{q_{s}}{q_{s,max}}<0.05$, the excess (E) regime as $\frac{q_{s}}{q_{s,max}}>0.95$, with the limitation (L) regime in between. Regime residence times are determined by registering the transitions of parcels between regimes; to reduce registration of short‐time, low amplitude transitions, a moving average filter with a window $\tau_{lag}=0.36$ s is applied, and transitions are only registered if the magnitude exceeds the regime boundary by 0.01; for example, a transition from excess to limitation is only registered for $\frac{q_{s}}{q_{s,max}}<0.94$, and the converse only for $\frac{q_{s}}{q_{s,max}}>0.96$. The regime transitions are summarized into regime residence time distributions, where trajectories through limitation are discriminated based on origin and destination, yielding four possibilities: “ELE”, “SLS”, “ELS”, “SLE”, codifying the regimes of origin, residence, destination, respectively. The average regime residence time is calculated as $\bar{\tau_{reg}}=\frac{\sum \tau_{reg}\cdot n_{\tau }}{\sum n_{\tau }}$ with $n_{\tau }$ the number of counts for residence time $\tau_{reg}$. Arc analysis is based on the movement of parcels with respect to a single threshold. Based on the structure of the lifelines, a threshold of $\frac{q_{s}}{q_{s,max}}=0.05$ is selected [7]; again, the duration between successive crossings of this threshold is registered, applying the same filtering steps as for regime analysis. For trajectories above the threshold, the maximum $\frac{q_{s}}{q_{s,max}}$ is logged; the maximum $\frac{q_{s}}{q_{s,max}}$ is correlated with the trajectory duration $\tau_{arc}$ to provide insight in the conditions registered along the trajectory. In contrast to prior FV‐RANS, the dynamic flow in LB‐LES means the regime layout in the simulations will be dynamic as well.

#### 2.3.5 Metabolic model coupling

The impact of extra‐cellular fluctuations on intra‐cellular pools, including the penicillin production rate $q_{p}$, is assessed as a post‐processing step. As in prior work [7], composite lifelines spanning 80 h of flow‐time under chemostat conditions are generated by back‐to‐back merging of the lifelines collected in the CFD simulations. Though this results in $q_{s}$‐jumps at the merging points, this very brief jump has no discernable impact on the long‐term metabolic response [7]. The 9‐pool model for penicillin production [52] is used to assess the metabolic response in MATLAB 2021b, assuming a fixed value for maximum glucose transport ($q_{E11,max}$) to mimic chemostat conditions. The response is averaged over 15 composite lifelines. Results are compared with the FV‐RANS simulation (average of 100 tracks [7]). For reference, the 9‐pool model is added in Appendix A. Currently, direct incorporation of a multi‐pool model into M‐STAR, to facilitate two‐way metabolic coupling [1, 2], is not yet possible. No fundamental issues are expected with such a coupling, as similar phenomena are already implemented for mass transfer in Lagrangian bubbly flows [32]. A high number of parcels may be needed to avoid (significant) gradients originating from the discretization of the biomass phase into parcels in combination with the high spatial resolution [53], however.

## 3 RESULTS AND DISCUSSION

### 3.1 Single impeller case

First, the single‐impeller case is discussed to judge hydrodynamic performance.

#### 3.1.1 Impeller discharge profiles

**FIGURE 1:**
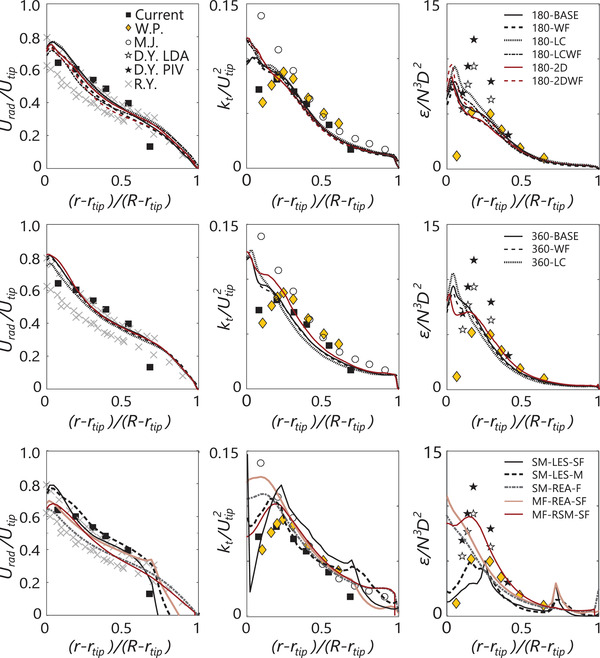
Impeller outflow profiles, quantified at *y* = T/3 ($\theta =45^{o}$, between baffles), compared with experimental data. Experimental data (time‐averaged): Haringa et al. (black boxes) [9], Murthy and Joshi (open circles) [41], Wu and Patterson (yellow diamonds) [38, 39], Ducci et al. (stars) [40], and the velocity summary by Ranade and Joshi (purple crosses) [36]. Top row: LB‐LES simulations (time‐averaged) with NX = 180. Middle row: LB‐LES simulations (time‐averaged) with NX = 360. Bottom row: Selected finite‐volume simulations from [9, 10]. SM‐LES‐SF: Sliding mesh LES (dynamic, time‐averaged, baffle plane), 10584k gridcells. SM‐LES‐M: Sliding mesh LES (dynamic, time‐averaged, baffle plane), 1997k grid cells. SM‐REA‐F: Sliding mesh, realizable k−ε (dynamic, time‐averaged), 5884k gridcells. MF‐REA‐SF: Multiple reference frame, realizable k−ε (static, baffle plane), 10584k. MF‐RSM‐SF: Multiple reference frame, Reynolds Stress Model (static, tangentially averaged), 10584k

In Figure 1, the time‐averaged radial velocity $U_{rad}$, turbulent kinetic energy $k_{t}$, and energy dissipation rate ε are plotted in the impeller discharge (between baffles). The radial velocity profiles lie perfectly in the range summarized by Ranade and Joshi [36]. The turbulent kinetic energy magnitude for ($r-r_{tip})/\left(R-r_{tip}\right)>0.2$ is well in line with the experimental data of Haringa et al. [9]; Wu and Patterson [38] as well as Murthy and Joshi [37] register somewhat higher values, although this may be a matter of experimental accuracy or subtle geometrical differences. Near the impeller tip, the simulations follow the data measured by Murthy and Joshi, who measured all three (fluctuating) velocity components whereas the other studies only measured $u_{rad}^{\prime }$ and $u_{ax}^{\prime }$, and assumed isotropy for $u_{\theta }^{\prime }$ – an assumption that breaks down near the impeller [26]. Most differences between simulation settings are also registered near the impeller, in particular at lower grid resolution. The low Co simulations as well as the simulations with a 2D impeller geometry register a somewhat higher $k_{t}$; use of wall functions has little impact. A similar tendency is observed for the profiles of ε, although the combination of the 2D impeller and wall functions leads to an even higher ε close to the impeller tip. A further look at flow behavior near the impeller tip is required to comment on the origin of these differences, which is out of the current scope. The magnitude of the peak in ε is reasonably predicted $\left(\frac{\varepsilon }{N^{3}D^{2}}\approx 10\right)$ compared to the measurements of Ducci et al. [40], except with for cases 180‐BASE and 180‐WF. However, the peak is located close to the impeller tip, whereas measurements put the location at $\left(r-r_{tip}\right)/\left(R-r_{tip}\right)=0.2$. Physically, this implies the energy transfer from the coherent trailing vortices to smaller turbulent structures can be improved in the simulations; there seems to be an early onset of substantial energy dissipation close to the impeller blades. It is possible this is observed due to the simple Smagorinsky subgrid model, relating local energy dissipation directly to the resolved‐scale strain rate, assuming equilibrium between the resolved and subgrid scale. Potentially, this can be improved with a more advanced subgrid model that accounts for turbulent kinetic energy, such as the dynamic kinetic energy subgrid model [54]. It must be noted that Ducci et al. measured 9 of the 12 mean squared velocity gradients required to determine ε and used isotropy assumptions for the others; as for $k_{t}$ this may impact on the experimentally determined values. Interestingly, in finite‐volume LES using ANSYS FLUENT a strong under‐prediction of ε was observed [10], particularly of the peak at $\left(r-r_{tip}\right)/\left(R-r_{tip}\right)=0.2$, but the location of the peak did match experiments. One potential origin of this difference is the usage of the dynamic Smagorinsky model of Germano et al. [55] in this prior work [10, 34]; a regular Smagorinsky simulation in FLUENT could provide insight, but the time requirements for this are strongly prohibitive. It is not expected that the notion that the FLUENT simulations were done with a 2‐impeller system makes a substantial difference due to the large separation between the impellers [56]; this is in agreement with the notion that the experimental data of Haringa et al. (two impellers) matches the other sources [36, 37, 38, 39, 40] (1‐impeller). Furthermore, it is supported by an additional 2‐impeller LB‐LES simulation (Appendix B).

#### 3.1.2 Power numbers

The overall energy dissipation is frequently lower than the power input from torque in CFD simulations, with under‐estimations of 69% being reported for scale‐resolved Detached Eddy Simulations (DES) [57]. As observed in Table 3, decent closure of the energy balance is observed currently, with the highest offsets (15% *higher* dissipation) observed using 2D sheet body internals. The power number is slightly under‐estimated compared to experimental data for Rushton turbines (Po=4.6−6.0) [58]; with $Po_{\tau }\approx 3.7-4.0$ and $Po_{\varepsilon }\approx 4.0-4.5$. An increase in the Smagorinsky parameter $C_{LES}$ may correct this offset [59], [60], although this is not consistent with DNS analysis [25]. As for the energy dissipation profiles, there is no substantial influence of spatial resolution, while a low Co leads to a higher power input and energy dissipation in the NX‐180‐cases. Wall functions slightly but consistently lower power input/dissipation; in line with the notion that LES without wall‐functions overestimates energy dissipation in the near‐wall region [49, 50].

**TABLE 3:** Global hydrodynamic performance for the different 1‐impeller cases

Case	$Po_{\tau }$	$Po_{\varepsilon }$	$\rho_{LB,min}$	$\rho_{LB,max}$
180‐BASE	3.87±0.07	4.01±0.08	0.994	1.004
180‐WF	3.69±0.09	3.81±0.11	0.994	1.004
180‐LC	4.44±0.10	4.65±0.11	1.000	1.000
180‐LCWF	4.23±0.13	4.44±0.14	1.000	1.000
180‐2D	3.94±0.13	4.51±0.11	0.994	1.004
180‐2DWF	3.84±0.07	4.01±0.08	0.994	1.004
360‐BASE	3.84±0.08	4.29±0.09	0.990	1.004
360‐WF	3.69±0.09	4.12±0.10	0990	1.004
360‐LC	3.89±0.08	3.97±0.08	1.000	1.000
360‐2D	4.14±0.11	4.81±0.13	0.991	1.004

Except for the lattice density $\rho_{LB},$ numbers are the mean ± one standard deviation. Left‐to‐right: Torque power number $Po_{\tau }$ dissipation power number $Po_{\varepsilon }$, minimum and maximum LB‐density $\rho_{LB}$.

Overall, the LB‐LES approach provides good quality results for a single‐phase stirred tank simulation, in agreement with previous studies. Compared to FV‐LES, more favorable characteristics in impeller discharge profiles are observed, especially regarding ε. The overall energy input is still somewhat under‐estimated, and the location of the peak in ε requires more attention, but for the purpose of resolving substrate gradients in the vessel bulk, the simulations are satisfactory. Due to the low degree of mesh dependency, it is expected that decent mixing simulations can be conducted with modest mesh resolutions.

### 3.2 Bioreactor case

Based on the single impeller results, a *2D* baffle/impeller geometry is used, wall functions are disabled, and as the lattice density fluctuations were well within 1%, a higher Courant number (Co = 0.075) is applied to reduce computation time. This may lead to a slight decrease in energy dissipation, but this is not considered a critical; the mixing performance is not substantially affected by under‐resolution of the energy dissipation rate in LES [10, 34]. Mixing behavior is quantified with a single probe, for comparison with prior work and experimental data. More detailed volumetric mixing was addressed by Fitschen et al. [61] showing very favorable mixing behavior compared to detailed photographic measurements.

#### 3.2.1 Mixing behavior

In a two‐Rushton system, flow compartments form around the individual impellers, with exchange between the compartments being rate limiting for mixing [9, 62]. Frozen‐flow (multiple‐reference frame) RANS simulations strongly underestimates this exchange large‐scale turbulence and the impact of macro‐instabilities are not captured [9, 10]. Dynamic sliding‐mesh RANS does capture macro‐instabilities but underestimates the turbulent component, whereas FV‐LES captures both influences. However, both sliding‐mesh RANS and FV‐LES are plagued by extensive computation times. In order to remedy inadequate inter‐compartment transport in steady‐state FV‐RANS, several authors tuned the turbulent Schmidt number, which relates turbulent species transport to the turbulent viscosity as $\sigma_{Sc}=\frac{\mu_{t}}{\rho D_{t}}$, down to $\sigma_{Sc}=0.2$, promoting “turbulent diffusion” over the inter‐compartment plane [15, 63, 64]. The drawback is that it also unnecessarily boosts mixing *inside* the compartments, while FV‐RANS with the default $\sigma_{Sc}=0.7$ show this is unnecessary [46]. Such boosting may hence reduce the accuracy of gradient predictions inside the compartments.

**FIGURE 2:**
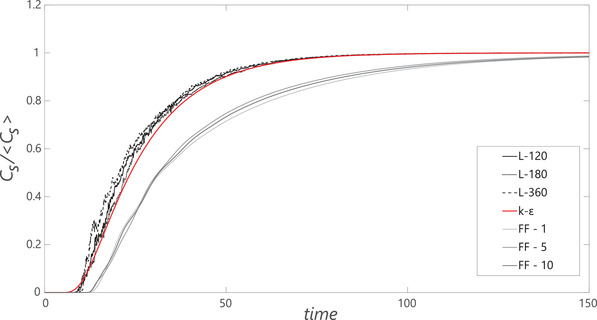
Mixing curves for the large‐scale bioreactor. Black curves: Dynamic simulations with three mesh resolutions. Gray curves: time‐averaged frozen flow (NX = 120) with three timescale‐factors. LB‐LES simulations are compared with prior frozen‐flow FV‐RANS simulations [3], shown in red

In Figure 2, the current dynamic LB‐LES simulations are compared with prior frozen‐flow FV‐RANS [3] with $\sigma_{Sc}=0.2$. Although the overall curves look very similar and exhibit nearly equal $\tau_{95}$ as well as $\tau_{circ}$ (Table 4), subtle differences can be identified. The slightly steeper slope of LES mixing curves indicates that mixing through the rate‐limiting inter‐impeller section by axial transport is somewhat faster; this is compensated by slightly faster intra‐compartment mixing due to the low $\sigma_{Sc}$ in the FV‐RANS simulations, yielding an overall similar circulation time, in agreement with experimental registration (unfortunately, no experimental registration of $\tau_{95}$ is available). In the LB‐LES simulations, inter‐compartment transport is correctly predicted by the dynamic macroscopic flow, requiring no ad‐hoc parameter tuning; similar to FV‐LES, but at much reduced computation time. The mixing curves with time‐averaged frozen‐flow in LB‐LES (cases FF‐1, 5, 10) exhibit much slower mixing, with no impact of the value of the Courant number. This is unsurprising; averaging the flowfield re‐introduces the issue of averaging out macro‐instabilities and dynamic turbulent exchange. In FV‐RANS, tuning $\sigma_{Sc}$ could boost transport, but no such parameter exists in the current approach. Due to the poor performance, further steps such as parcel tracking are not conducted in the frozen flowfield. Leveraging frozen flow in LB‐LES would require the option to impose the impact of turbulence based on the local values of $k_{t}$ and ε, similar to FV‐RANS.

**TABLE 4:** Mixing time registration for various cases

Case	$\tau_{circ}$ [s]	$\tau_{95}$ [s]
L‐120	21.7	58.8
L‐180	20.5	60.9
L‐300	21.5	57.7
L‐FF1	30.9	110.9
L‐FF5	39.5	102.4
L‐FF10	29.4	106.8
FV‐RANS	18.2	60.9
Industrial	19.3	n/a

The circulation time is defined as twice the time lag between tracer injection at the top, and 5% saturation of the probe on the bottom. Both mixing and circulation time are given in seconds.

#### 3.2.2 Substrate gradients

**FIGURE 3:**
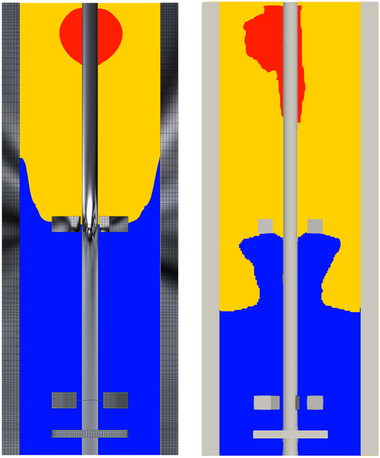
Comparison of the observed substrate gradient, expressed in terms of uptake saturation ($C_{s}/\left(C_{s}+K_{s}\right)$) and colored by metabolic regimes [3]: red: excess, yellow: limitation blue: starvation. Left: FV‐ (steady‐state), right: LB‐LES (time‐averaged)

The substrate gradient is recorded with a constant substrate feed inserted in the top, combined with volumetric Monod kinetics. As the mixing dynamics showed no dependence on resolution, the kinetic‐ and parcel‐tracking simulations, requiring longer flow‐timespans, are conducted on the crudest lattice (NX = 120). The concentration gradient acquired by FV‐RANS and LB LES is compared in Figure 3. Because the substrate concentration spans many orders of magnitudes, the time‐averaged gradient is plotted in terms of the regimes outlined in Section 2.3.4. Due to the dynamic nature of LB‐LES, the instantaneous substrate distribution (and hence regime map) are in reality dynamic in this simulation. As expected from mixing, the gradient is similar between FV‐RANS and LB‐LES, but there are notable differences. Somewhat stronger (axial) convection is predicted in LB‐LES, leading to the excess regime being stretched due to the stronger vertical flow. The narrower excess regime on the right‐hand side is due to absence of a symmetry assumption in the LB‐LES simulation, while for FV‐RANS, a $180^{o}$ segment of the reactor is modeled, inducing symmetry in the excess regime. The most notable difference, also induced by faster axial transport in LB‐LES, is the different limitation‐starvation regime boundary around the top impeller. In the FV‐RANS simulation, starvation is observed in the impeller discharge stream, whereas in the LB‐LES simulation, the stronger axial flow in the top segment leads to a stronger supply of glucose into the discharge stream, which is not instantly depleted upon contact with the glucose‐lean flow from the reactor bottom. Even though difference in $\tau_{95}$ is minor between FV‐RANS and LB‐LES, the slight differences in flow‐patterns lead to a substantially smaller starvation regime and larger limitation regime (Table 5). These changes in regime layout will impact regime residence times, considering the location on the intersection of re‐circulation loops, and may propagate to differences in the metabolic response.

**TABLE 5:** Fractional distribution of the regimes in LB‐LES versus FV‐RANS, registered from the Lagrangian point of view

Case	LB‐LES	FV‐RANS
Excess (E)	7.8	7.1
Limitation (L)	43.7	34.7
Starvation (S)	48.5	58.2

#### 3.2.3 Analysis of organism lifelines

**FIGURE 4:**
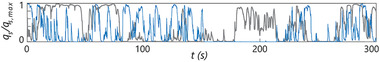
Example of a lifeline collected in this study. Blue: LB‐LES. Gray: FV‐RANS

**FIGURE 5:**
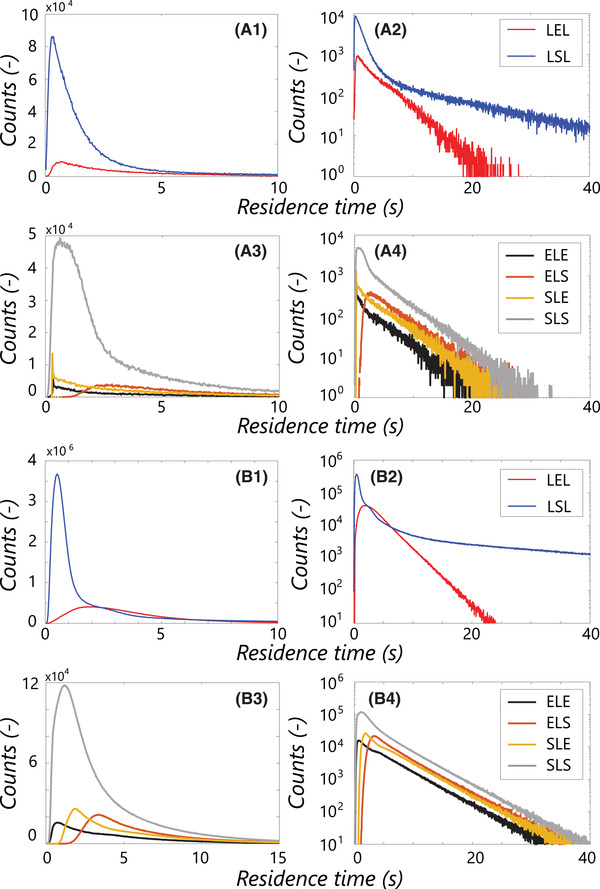
(A1–A4) Non‐normalized regime distributions in the LB‐LES simulations. (A1 and A2) distributions of residence time in the excess (LEL) and starvation (SLS) regime (A1: regular y‐axis, A2: logarithmic y‐axis). (A3 and A4) distributions for the four transition patterns through the limitation regime (A3: regular y‐axis, A4: logarithmic y‐axis). B1‐B4: The same figures for the FV‐RANS simulation reported in [3]. The difference in the number of counts between (A) and (B) is due to the different number of particles $N_{p}$ and tracking duration between the simulations

Parcels are tracked for 2000 s, of which the first 100s are discarded for initial distribution. The acquired lifelines show roughly similar qualitative features to those collected with FV‐RANS [3] (see Figure 4), but appear a more “noisy” due to the higher spatial resolution and dynamics in the concentration gradient. Quantitatively, application of regime analysis does reveal differences, following from differences in the regime map, while the dynamics in flowfield in LB‐LES may also play a role. Figure 5, A1–A4 shows the regime residence time distribution registered LB‐LES. Although more noisy than the FV‐RANS results (Figure 5B1–B4) due to the lower $N_{p}$ (leading to lower absolute counts), many features in the distributions are similar between LB‐LES and FV‐RANS.

The additional fluctuations originating from the flow dynamics do not seem to have a strong impact on the observed transitions, likely because these are modest in magnitude; the differences between LB‐LES and FV‐RANS mainly arise from changes in large‐scale mixing behavior. Residence times in the excess regime are similar, evidenced both by the plot, and the nearly equal mean residence time (Table 6). In limitation, the residence times are slightly lower for all four trajectories, matching the observation of faster axial transport in Figure 3, and reflected by maximum residence times in the range of 20–30 s (Figure 5A3–A4), whereas 30–40 s was registered with FV‐RANS. Qualitatively, the curves look similar between LB‐LES and FV‐RANS, with for example, the delayed peak in the ELS‐distribution and the large peak in the SLS distribution (Figure 5A3) observed in both, the latter caused by parcels that briefly pass through the top‐impeller discharge stream before returning to the bottom. In the starvation‐regime, two circulation modes, represented by two different slopes in the distribution, are observed in Figure 5A2. The second slope, representing long residence times in starvation, originates from parcels stuck in the bottom impeller circulation. However, the notion that the top‐impeller discharge stream is, on average, in the limitation regime in LB‐LES does induce differences. The first LSL peak, representing parcels that only very briefly pass through starvation, is more prominent in LB‐LES, giving a much lower mean regime residence time (Table 6). Furthermore, the slope of the long‐residence time SLS distribution is steeper, because parcels that move close to the top impeller are likely to briefly hit limitations conditions, giving fewer very long uninterrupted starvation exposures.

**TABLE 6:** Average regime residence times (in seconds) in the LB‐LES and FV‐RANS simulations, registered per transition pattern

Transition pattern	$\tau_{reg}$ [s], LB‐LES	$\tau_{reg}$ [s], FV‐RANS
LEL	3.63	3.65
SLS	4.38	9.37
ELE	4.02	4.67
ELS	5.96	6.45
SLE	5.25	5.39
SLS	3.11	3.77

The pattern is codified in the name as ELS = residence time in L(imitation) for a parcel coming from (E)xcess and moving to (S)tarvation.

**FIGURE 6:**
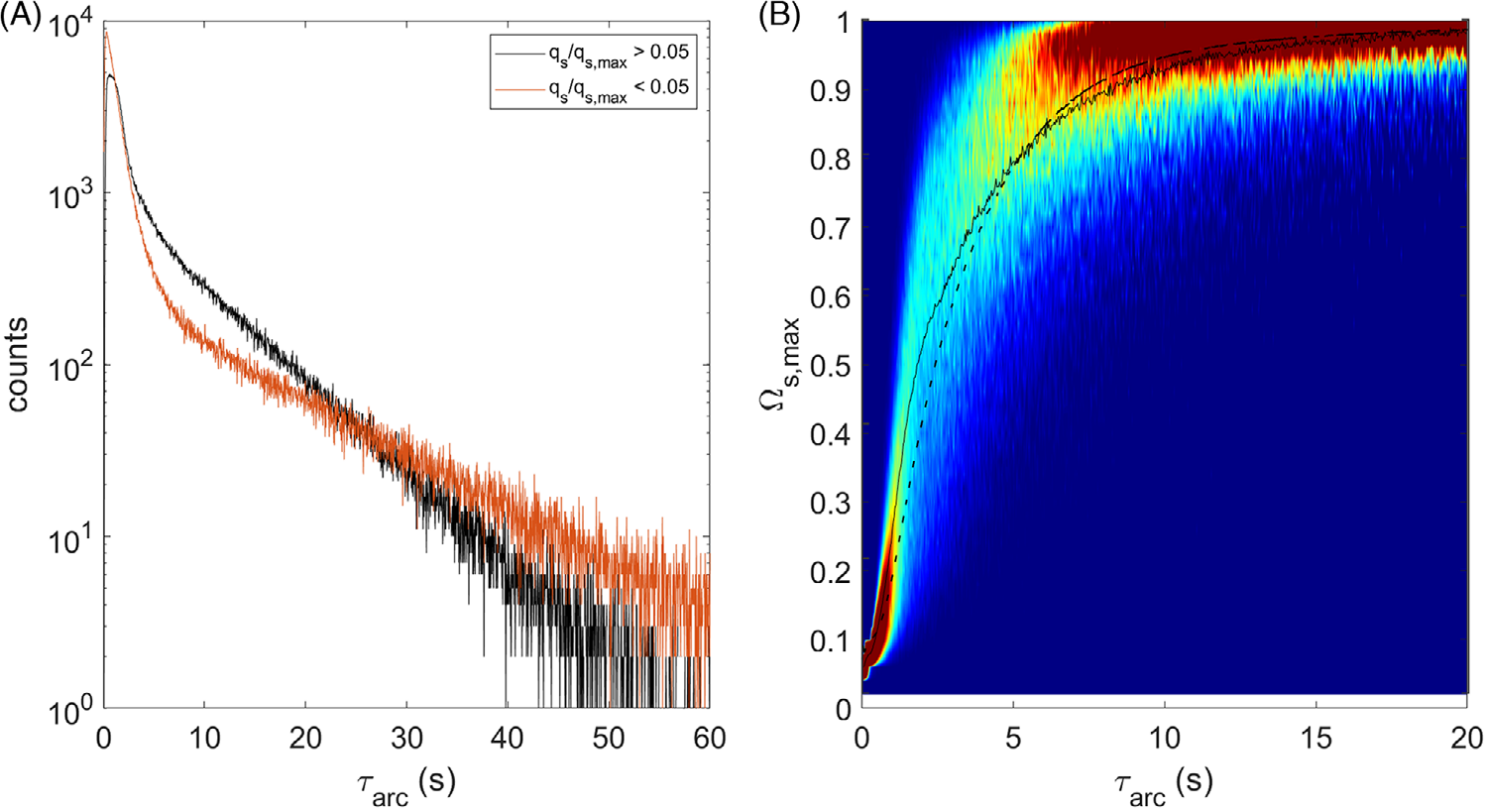
(A) Arc‐time distributions for $\frac{q_{s}}{q_{s,max}}\leq 0.05$ (gray) and $\frac{q_{s}}{q_{s,max}}>0.05$. (B) Arc magnitude as a function of arc time for $\frac{q_{s}}{q_{s,max}}>0.05$. Colors indicate bin fraction (normalized per timestep). Solid line: average magnitude versus $\tau_{arc}$ for LB‐LES. Dashed line: average magnitude versus $\tau_{arc}$ for FV‐RANS

The lifelines are next subjected to arc analysis, using $q_{s}/q_{s,max}=0.05$ as a threshold. As for regime analysis, the qualitative features agree well between the simulation methodologies, but quantitatively some differences are observed. As $\tau_{arc}$ for starvation is equal to the residence time in the starvation regime, also here the frequency changes in short and long starvation events are noted (Figure 6A). There is more scatter in the contour plot relating arc duration with magnitude (Figure 6B), which in part is caused by the lower number of parcels, but likely also by the dynamic nature of the simulation leading to somewhat more variation in local the conditions observed along parcel trajectories, caused not just by variations in the trajectory itself, but also by variations in the underlying fluid flow, and consequently, concentration gradients.

#### 3.2.4 Intra‐cellular pool response

**FIGURE 7:**
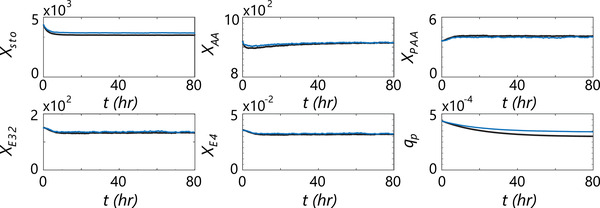
Metabolic response computed by the 9‐pool model for penicillin production . Black: FV‐RANS (from Ref. [7]), average of 100 composite particle tracks. Blue: LB‐LES (current work), average of 10 composite particle tracks. Pool $X_{E11}$ is not displayed as it is frozen in the current setup, pools $X_{gly}$ and $X_{ATP}$ are not displayed because their dynamics are fast compared to the timescale of the whole process

The intra‐cellular pool response is computed as a post‐processing step, by feeding 15 extended lifelines into the 9‐pool penicillin production model [52] (details in Appendix A). The response for 6 intra‐cellular pools is reported in Figure 7, compared to the response for the FV‐RANS model (case TU‐B from [7]). The pools are initialized assuming ideal mixing under chemostat conditions; after ca. 80 h under non‐ideal mixing conditions, new steady‐state values for the intra‐cellular pools are established. While a similar trend can be observed for the penicillin production rate $q_{p}$, the final value of $q_{p}$ is higher in LB‐LES; a $q_{p}$ loss of 22% is observed, compared to 32% in FV‐RANS. This is consistent with the notion that the LB‐LES simulation is slightly better mixed, as evidenced from the regime distribution in Table 6, and therefore a lesser impact of heterogeneous conditions on the penicillin production rate is expected.

### 3.3 Computation time analysis

There are considerable differences between FV‐RANS and LB‐LES in time requirements and simulation set‐up. To provide a comparison, several FV‐RANS/LES simulations from prior work [9, 10] were partially re‐run to estimate the runtime on contemporary hardware, using a 12‐core XEON‐W2265 desktop with 10 cores dedicated to the simulation. The run‐time per second flow‐time is compared GPU‐based LB‐LES simulations on the same desktop. As such, the comparison provides insight in the computational gain running on a high‐end desktop. Table 7 shows the advantage of LB‐LES: the high resolution case (50M lattice points, ∼1000 s/s run/flowtime) runs approx. 10x faster than the crudest dynamic (sliding mesh, 2.0M gridcells) FV‐RANS case, and 200× faster than the 10M gridcell FV‐LES case. The coarse LB‐LES case (5.8M lattice points) runs ca. 10× faster *dynamically* than *frozen‐flow* FV‐RANS of similar resolution, aside from the time needed to converge the flow. FV‐RANS/LES does stand to gain from using a computing cluster, but even when assuming perfect scaling down to 10 000 gridcells/core, the computation time would be 491 s/s and 1865 s/s for sliding mesh FV‐RANS and FV‐LES, respectively – of the same order as LB‐LES case 360‐Base, but on substantially more expensive hardware. Furthermore, the LB‐LES simulations may equally benefit from a GPU cluster. Furthermore, in FV‐RANS, generation of a good quality mesh is required as pre‐processing, taking between an hour and a week, depending on geometric complexity, the software, and the experience of the user. In LB, use of a homogeneous lattice means no meshing is required, substantially reducing setup time.

**TABLE 7:** Runtime requirements for a selection of cases, small tank case

Case(measured)	Grid	Δt[μs/iter]	Run‐time [s/s]	Hardware
180‐Base	5.8M	53.0	85	GPU‐RTX3090
360‐Base	46.7M	26.5	1003	GPU‐RTX3090
Case (measured)	Grid	Δt[μs/iter]	Run‐time/flow time [s/s]	Hardware
MF‐REA‐F (frozen flow)	5.8M	5000/5	34642 s (flow convergence) 886 s/s (frozen flow mixing)	W2265 @10core
SM‐REA‐M	2.0M	3333/50	9818	W2265 @10core
SM‐LES‐SF	10.6M	1667/50	197643	W2265 @10core

The top four cases are LB‐LES simulations conducted in the current work. The bottom three cases are finite volume simulations. Grid represents total no. lattice points (LB) or grid‐cells (FV). For FV‐cases, besides the timestep size, max. iterations (“iter”) are displayed. Case MF‐REA‐F: Multiple reference frame, realizable k−ε, with frozen flow mixing. SM‐REA‐M: Sliding mesh, realizable k−ε, dynamic. SM‐LES‐SF finite volume, sliding mesh Large Eddy Simulation, dynamic. For simulation MF‐REA‐F, steady‐state flow was computed with <0.1% variation in mean ε over 50 successive iterations as convergence criterion. The flow was subsequently frozen, and only species transport was solved dynamically. The other simulations are analogous in setup to prior work [10].

## 4 CONCLUDING REMARKS

Lattice‐Boltzmann Large Eddy Simulations (LB‐LES) have successfully been applied to study substrate heterogeneity in an industrial scale bioreactor from the microbial perspective. First, the performance of LB‐LES in single phase hydrodynamic simulations has been confirmed by comparing radial velocity, turbulent kinetic energy and energy dissipation the impeller discharge stream with prior experimental results, observing good matches for the radial velocity and turbulent kinetic energy. While the energy dissipation peak in the impeller outflow was not reproduced, performance was much better than for previous FV‐LES. The global energy dissipation was somewhat under‐estimated, observing $Po_{\varepsilon }\approx 4-4.5$ while ∼5 is expected, but this under‐estimation is minor compared to FV‐LES. Mixing behavior in a large‐scale bioreactor was very similar between LB‐LES and prior Finite Volume Reynolds‐Averaged Navier‐Stokes (FV‐RANS) simulations, but LB‐LES does not require ad‐hoc tuning of the turbulent Schmidt number as the fully dynamic flow captures all relevant flow‐features for mixing. Nevertheless, LB‐LES runs significantly faster than dynamic FV‐RANS simulations, and is even competitive with frozen‐flow RANS in terms of computation time. For the first time, microbial lifelines were acquired in an LB‐LES bioreactor simulation; the collected lifelines have been scrutinized with established methods for lifeline analysis. There were some quantitative differences; the stronger axial transport in LB‐LES led to a larger limitation and smaller starvation regime, reflected in reduced regime residence times in the starvation regime compared to FV‐RANS. The average residence time under limitation conditions was also slightly reduced, due to faster axial transport in the reactor top. When coupled with the 9‐pool penicillin production model, a milder decrease in penicillin production rate was predicted compared to FV‐RANS, consistent with the observed changes in regime distribution.

Although differences exist, with appropriate settings, both FV‐RANS and LB‐LES are well suited to conduct lifeline analysis for downscaling, estimation of productivity losses, and in‐silico optimization of bioreactor design. However, LB‐LES has strong performance advantages: it provides a dynamic flowfield and substrate gradient, requires no parameter tuning for multi‐impeller mixing, yields a favorable computation time and requires no meshing. This makes LB‐LES a highly promising tool for design screening and optimization.

## CONFLICT OF INTEREST

The author declares there are no conflicts of interest.

## NOMENCLATURE

**Table:** 

Symbol	Unit	Description
C	[m]	Impeller off‐bottom clearance
ΔC	[m]	Impeller mutual clearance
$C_{LES}$	−	Smagorinsky constant (LES)
$C_{s}$	[mol/kg]	Substrate concentration
$C_{x}$	[g/kg]	Biomass concentration (Eulerian)
Co	−	Courant number
$d_{s}$	[m]	Shaft diameter
D	[m]	Impeller diameter
D	[m^2^/s]	Diffusion coefficient (glucose in water)
$F_{s}$	[mol/s]	Substrate feed rate (general)
$H_{l}$	[m]	Liquid filled height
$k_{t}$	[m^2^/s^2^]	Turbulent kinetic energy
$K_{s}$	[mol/kg]	Substrate affinity constant
$N_{p}$	−	Total number of parcels
N	[RPM]	Agitation rate
NX	−	Grid divisions, x‐direction
$Po_{\tau }$	−	Power number, torque
$Po_{\varepsilon }$	−	Power number, dissipation
$q_{s}$	[mol_s_/g_dw_/s]	Specific uptake rate, substrate
$q_{s,max}$	[mol_s_/g_dw_/s]	Maximum specific uptake rate, substrate
r	[m]	Radial coordinate
$r_{tip}$	[m]	Impeller radius
R	[m]	Tank radius
t	[s]	Time (general)
Δt	[s]	Timestep size (general)
$\Delta t_{p}$	[s]	Timestep size, parcel tracking
$\Delta t_{write}$	[s]	Timestep size, parcel data writing
T	[m]	Tank diameter
$u^{\prime }$	[m/s]	Fluctuating velocity
$U_{rad}$	[m/s]	Radial velocity
$v_{tip}$	[m/s]	Impeller tip speed
V	[m^3^]	Volume
Δx	[m]	Grid spacing
$X_{pool}$	$\frac{\mu \mathrm{mol}}{g_{\mathrm{dw}}}$ (metabolite) ‐ (enzyme)	Intracellular pool size
y	[m]	Axial coordinate

### Greek symbols

**Table:** 

Symbol	Unit	Description
ε	[m^2^/s^3^]	Turbulent energy dissipation rate
$\rho_{l}$	[kg/{m^3^]	Liquid density
$\rho_{LB}$	−	Lattice density
$\mu_{l}$	[Pa s]	Liquid viscosity
$\Omega_{s,max}$	−	Arc magnitude
$\sigma_{Sc}$	−	Turbulent Schmidt number
$\tau_{95}$	[s]	Mixing time (95%)
$\tau_{circ}$	[s]	Circulation time
$\tau_{arc}$	[s]	Arc duration
$\bar{\tau_{reg}}$	[s]	Regime residence time, average

## Supporting information

SUPPORTING INFORMATION
