# Supplementary material for: An analysis of organism lifelines in an industrial bioreactor using Lattice‐Boltzmann CFD
Source: Eng Life Sci. 2022 Mar 16;23(1):e2100159. doi: 10.1002/elsc.202100159 (PMC9815090; doi:10.1002/elsc.202100159)
Supplement: Supplementary file 1 — SUPPORTING INFORMATION [file ELSC-23-e2100159-s001.pdf]

Appendix A. 9-pool model

The model stoichiometry is given in table A.1, the kinetic equations in table A.2, and model parameters in A.3. As the ATP balance in the original model was destabilized by rapid concentrations in extracellular glucose, the intra-cellular ATP concentration (X_{ATP}) was assumed to be correlated with the concentration of intra-cellular glycolytic intermediates [1] as $X_{ATP} = A \cdot X_{gly}^3/(X_{gly}^3 + B^3)$. In previous work, $A = 8.5$ µmol/g$_{dw}$ and $B = 10.5$ µmol/g$_{dw}$ were determined, which was used in our simulations.

Table A.1: Stoichiometric matrix of the metabolically structured kinetic model (in mol/mol, from: Tang et al. [2]). X_{bio} is the biomass pool, *gluc* the extra-cellular glucose pool.

Pool	v_{11}	v_{12}	v_{13}	v_{21}	v_{22}	v_{31}	v_{32}	v_{33}	v_{41}	v_{42}	v_d
Glyc.	6	−1	−0.578	−1	0	0	0	−4.81	−1.07	1	0
AA.	0	1	−0.5	0	0	0	0	−6.25	0	0	0
Sto.	0	0	0	0	0	0	0	0	1	−1	
ATP	−2	−0.65	−1.037	4.43	−1	0	−2	−8	−0.167	−0.167	0
PAA	0	0	0	0	0	1	−1	−1	0	0	0
Gluc.	−1	0	0	0	0	0	0	0	0	0	0
X_{bio}	0	0	1	0	0	0	0	0	0	0	−1

Table A.2: Kinetic equations of the $9-pool$ model.

Reaction	Kinetics	Eq.
Transporter capacity	$\frac{dX_{E,11}}{dt} = q_{E,11,max} \cdot \frac{((\mu+\mu_0)/k_{11})^5}{1+((\mu+\mu_0)/k_{11})^5} - (\mu + k_{dE,11})X_{E,11}$	$dv_{X,11}$
Carbon uptake	$v_{11} = k_{E,11}X_{E,11}\frac{C_s}{C_s+K_{s,11}}$	v_{11}
Amino Acid synthesis	$v_{12} = v_{12,max} \cdot \frac{X_{gly}^2}{K_{gly,12}^2+X_{gly}^2} \cdot \frac{K_{AA,12}^2}{K_{AA,12}^2+X_{AA}^2} \cdot \frac{X_{ATP}^3}{K_{ATP,12}^3+X_{ATP}^3}$	v_{12}
Growth	$v_{13} = v_{13,max} \cdot \frac{X_{gly}^2}{K_{gly,13}^2+X_{gly}^2} \cdot \frac{X_{AA}^2}{K_{AA,13}^2+X_{AA}^2} \cdot \frac{X_{ATP}^3}{K_{ATP,13}^3+X_{ATP}^3}$	v_{13}/μ
ATP production	$v_{21} = v_{21,max} \cdot \frac{X_{gly}^3}{K_{gly,21}^3+X_{gly}^3} \cdot \frac{K_{ATP,21}^4}{K_{ATP,21}^4+X_{ATP}^4}$	v_{21}
Maintenance	$v_{22} = m_{ATP,22}$	v_{22}
PAA import	$v_{31} = k_{perm,31} \cdot a_{cell} \cdot \left(\frac{X_{PAA}/2.5}{1+10^{pH,ext-pK}} - \frac{C_{PAA} \cdot \rho_{broth}}{1+10^{pH,int-pK}}\right)$	v_{31}
PAA export capacity	$\frac{dX_{E,32}}{dt} = \alpha_{32} + \beta_{32} \cdot \mu - k_{dE,32} \cdot X_{E,32} - \mu \cdot X_{E,32}$	$dv_{X,32}$
PAA export	$v_{32} = X_{E,32} \cdot X_{PAA} \cdot M_X \cdot 10^{-6}$	v_{32}
Pen-G production capacity	$\frac{dv_{33}}{dt} = \frac{\beta_{33} \cdot \mu}{1+(X_{gly}/K_{gly,33})^{m33}} - (k_{dE,33} + \mu) \cdot v_3 3$	v_{33}/q_p
Storage capacity	$\frac{dX_{E,4}}{dt} = \alpha_4 + \beta_4 \cdot \mu - k_{dE,4} \cdot X_{E,4} - \mu \cdot X_{E,4}$	$dv_{X,4}$
Carbon storage	$k_{41} \cdot X_{E,4} \cdot \frac{C_s}{C_s+K_{s,41}} \cdot \left(1 + 2 \cdot \frac{C_s}{C_s+K_{s,42}}\right) \cdot \frac{K_{sto,41}}{X_{sto}+K_{sto,41}}$	v_{41}
Storage release	$k_{42} \cdot X_{E,4} \cdot \frac{K_{s,42}}{C_s+K_{s,42}} \cdot \left(1 + 2 \cdot \frac{K_{s,41}}{C_s+K_{s,41}}\right) \cdot \frac{X_{sto}^2}{X_{sto}^2+K_{sto,42}^2} \cdot \frac{X_{ATP}^2}{X_{ATP}^2+K_{ATP,42}^2}$	v_{42}
Death rate	$v_{d,m}$ (set 0, because C_x is fixed)	v_d

Table A.3: Parameters of the $9 - pool$ model.

Param.	Value	St. Err.	Unit
$q_{E,11,max}$	$6.5 \cdot 10^{-2}$	$4.41 \cdot 10^{-3}$	$U/C\text{mol}_x/\text{h}$
μ_0	$5.5 \cdot 10^{-2}$	$1.03 \cdot 10^{-3}$	h^{-1}
k_{11}	0.10	$1.16 \cdot 10^{-3}$	h^{-1}
$k_{dE,11}$	$1.46 \cdot 10^{-2}$	$6.55 \cdot 10^{-3}$	h^{-1}
$k_{E,11}$	0.26	$1.54 \cdot 10^{-2}$	mol_s/U
$k_{s,11}$	$7.8 \cdot 10^{-6}$	$1.54 \cdot 10^{-2}$	mol/kg
$v_{12,max}$	0.18	$7.74 \cdot 10^{-2}$	$\text{mol}/C\text{mol}_x/\text{h}$
$K_{gly,12}$	31.38	4.91	$\mu\text{mol/g}_{dw}$
$K_{AA,12}$	870.23	69.6	$\mu\text{mol/g}_{dw}$
$K_{ATP,12}$	2.01	0.26	$\mu\text{mol/g}_{dw}$
$v_{13,max}$	0.32	$9.92 \cdot 10^{-2}$	$\text{mol}/C\text{mol}_x/\text{h}$
$K_{gly,13}$	38.54	9.25	$\mu\text{mol/g}_{dw}$
$K_{AA,13}$	757.81	128.83	$\mu\text{mol/g}_{dw}$
$K_{ATP,13}$	1.95	0.24	$\mu\text{mol/g}_{dw}$
$v_{21,max}$	0.35	$9.45 \cdot 10^{-2}$	$\text{mol}/C\text{mol}_x/\text{h}$
$K_{gly,21}$	25.64	5.58	$\mu\text{mol/g}_{dw}$
$K_{ATP,21}$	6.01	0.66	$\mu\text{mol/g}_{dw}$
$m_{ATP,22}$	$3.3 \cdot 10^{-2}$	$1.2 \cdot 10^{-2}$	$\text{mol}/C\text{mol}_x/\text{h}$
$k_{perm,31}$	$1.62 \cdot 10^{-2}$	$3.74 \cdot 10^{-3}$	m/h
a_{cell}	56.00	–	$\text{m}^2/C\text{mol}_x$
α_{32}	0	0	h^{-2}
β_{32}	$1.56 \cdot 10^{3}$	218.4	h^{-1}
$k_{dE,32}$	0.35	$4.90 \cdot 10^{-2}$	h^{-1}
β_{33}	$6.5 \cdot 10^{-4}$	–	$\text{mol}/C\text{mol}_x/\text{h}$
$k_{dE,33}$	$1.47 \cdot 10^{-2}$	$1.2 \cdot 10^{-3}$	h^{-1}
$K_{33,gly}$	30.76	1.58	$\mu\text{mol/g}_{dw}$
m_{33}	6.00	–	–

Table A.4: Parameters of the $9-\textit{pool}$ model (continued).

Param.	Value	St. Err.	Unit
α_4	$8.01 \cdot 10^{-4}$	$1.68 \cdot 10^{-4}$	mol/Cmol$_x$/h^2
β_4	0.289	$1.49 \cdot 10^{-2}$	mol/Cmol$_x$/h
$k_{dE,4}$	0.29	$1.23 \cdot 10^{-2}$	h^{-1}
k_{41}	1.01	0.1	mol/mol
$K_{s,41}$	10^{-8}	$1.33 \cdot 10^{-8}$	mol/kg
$K_{sto,41}$	$4.25 \cdot 10^3$	$9.32 \cdot 10^2$	μmol/g$_{dw}$
k_{42}	3.99	0.48	mol/mol
$K_{s,42}$	10^{-4}	$1.37 \cdot 10^{-4}$	mol/kg
$K_{sto,42}$	$7.99 \cdot 10^3$	$1.07 \cdot 10^3$	μmol/g$_{dw}$
$K_{ATP,42}$	6.48	0.9	μmol/g$_{dw}$
pH_{int}	2.20	–	–
pH_{ext}	6.50	–	–
pK_{PAA}	4.31	–	–
$v_{d,m}$	$5 \cdot 10^{-3}$	–	h^{-1}
$M_{w,bio}$	28.05	–	g$_{dw}$/Cmol

Appendix B. Simulation of a 2-impeller stirred tank

To verify the assumption that impeller discharge profiles between in a 1-impeller Rushton tank are similar to those in a 2-impeller Rushton tank (at one tank diameter spacing [3]), an additional simulation with 2 impellers was conducted, following the geometry of Jahoda et al. [4], and using the same simulation settings as the 1-impeller simulations reported in this work (NX = 180). The results, shown in fig. B.1 confirm the hypothesis; some minor differences are observed, predominantly a slightly lower radial velocity, but overall, the discharge profiles follow the same trend. As such, we deem the comparison between 1-impeller simulations and (some) 2-impeller kinetic energy profiles/FV-RANS results (sec. 3.1.2) valid.

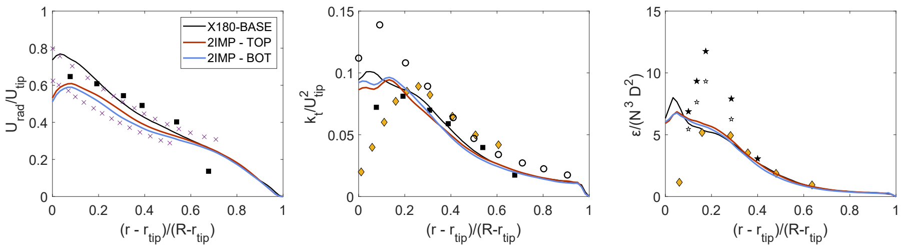

Figure B.1: Additional simulation of a 2-impeller stirred vessel (dimensions of [4] to confirm similarity in impeller discharge stream characteristics compared to the 1-impeller simulation. Quantified at $y = T/3$ (bottom) and $y = T + T/3$ (top) acquired in the between-baffle plane, compared with experimental data. Experimental data (time-averaged): Haringa et al. (black boxes) [5], Murthy and Joshi (open circles) [6], Wu and Patterson (yellow diamonds) [7, 8], Ducci et al. (stars) [9] and the velocity summary by Ranade and Joshi (purple crosses) [10].

References

[1] C. Haringa, W. Tang, G. Wang, A. T. A. Deshmukh, W. A. W. van Winden, J. Chu, W. W. M. van Gulik, J. J. J. Heijnen, R. R. F. Mudde, H. J. H. Noorman, Computational fluid dynamics simulation of an industrial P. chrysogenum fermentation with a coupled 9-pool metabolic model: To-

wards rational scale-down and design optimization, Chemical Engineering Science 175 (2018) 12–24.

[2] W. Tang, A. A. T. Deshmukh, C. Haringa, G. Wang, W. van Gulik, W. van Winden, M. Reuss, J. J. Heijnen, J. Xia, J. Chu, H. H. J. Noorman, A 9-pool metabolic structured kinetic model describing days to seconds dynamics of growth and product formation by Penicillium chrysogenum, Biotechnology and Bioengineering 114 (2017) 1733–1743.

[3] V. Hudcova, V. Machon, A. W. Nienow, Gas-liquid dispersion with dual Rushton impellers, Biotechnology and Bioengineering 34 (1989) 617–628.

[4] M. Jahoda, M. Moštěk, A. Kukuková, V. Machoň, CFD Modelling of Liquid Homogenization in Stirred Tanks with One and Two Impellers Using Large Eddy Simulation, Chemical Engineering Research and Design 85 (2007) 616–625.

[5] C. Haringa, R. Vandewijer, R. F. Mudde, Inter-compartment interaction in multi-impeller mixing: Part I. Experiments and multiple reference frame CFD, Chemical Engineering Research and Design 136 (2018) 870–885.

[6] B. Murthy, J. Joshi, Assessment of standard , RSM and LES turbulence models in a baffled stirred vessel agitated by various impeller designs, Chemical Engineering Science 63 (2008) 5468–5495.

[7] H. Wu, G. Patterson, Laser-Doppler measurements of turbulent-flow parameters in a stirred mixer, Chemical Engineering Science 44 (1989) 2207–2221.

[8] H. Wu, G. K. Patterson, M. Van Doorn, Distribution of turbulence energy dissipation rates in a Rushton turbine stirred mixer, Experiments in Fluids 8 (1989) 153–160.

[9] A. Ducci, M. Yianneskis, Direct determination of energy dissipation in stirred vessels with two-point LDA, AIChE Journal 51 (2005) 2133–2149.

[10] V. V. Ranade, J. B. Joshi, Flow generated by a disc turbine. II: Mathematical modelling and comparison with experimental data, Chemical engineering research design 68 (1990) 34–50.
